# Protocol for meta-research on the evidence informing micronutrient dietary reference intakes for pregnant and lactating women

**DOI:** 10.12688/gatesopenres.13199.1

**Published:** 2020-11-13

**Authors:** Siran He, Kevin C. Klatt, Ali Rahnavard, Matthew D. Barberio, Alison D. Gernand, Emily R. Smith

**Affiliations:** 1 Milken Institute School of Public Health, The George Washington University, Washington, DC, 20052, USA; 2 USDA Children's Nutrition Research Center, Baylor College of Medicine, Houston, TX, 77030, USA; 3 Pennsylvania State University, University Park, PA, 16802, USA

**Keywords:** Nutrition reference value, dietary reference intake, meta-science, inclusion, pregnancy, lactation

## Abstract

Nutrient reference values are important parameters that guide nutrition and public health work globally. Micronutrient requirements during the peri-conception period are generally increased, which is essential in ensuring maternal, fetal, and neonatal health. Nevertheless, the current dietary reference intakes (DRIs) may be limited in terms of the methods used and the populations included, particularly the DRIs for pregnancy and lactation. In this proposed review, we will examine the methods (rigor of design, utilization of molecular methods, and presence of modern methods) and the population (inclusion of women, and in particular, pregnant and lactating people) in the studies used to inform the current DRIs. We will apply meta-science methods to this review, which involves formally reviewing the current evidence, and identifying opportunities to improve how we fund, perform, evaluate, and incorporate nutrition science into public health programs for better outcomes.

## Introduction

### Rationale

Nutrient reference values (NRVs) play an essential role in promoting the health and well-being of a population. Conceptually, NRVs are meant to assess the risk of deficiency and inadequacy while avoiding excessive intakes for healthy populations, and to be utilized in planning adequate diets for individuals and populations. The key components of NRVs are the population average requirement (AR) and the safe upper intake level (UL) ^1^. In the 1990s, a group of experts proposed a then novel approach for setting new nutrient intake recommendations. These dietary reference intake (DRI) values were initially adopted in the US, in collaboration with Canada and the UK ^1^.

The World Health Organization (WHO) also provides NRVs (for vitamins and minerals) in a 2004 document, which is based on a 1998 convening joining the WHO and the Food and Agriculture Organization (FAO) of the United Nations ^2^. However, the DRIs are more widely used. This review will focus on the DRIs developed by the US, UK, and Canada, and not the WHO NRVs, for two main reasons. First, the NRVs set forth by WHO/FAO, despite considering several contextual factors, have not been recently updated for micronutrients. While a few important updates have been made to the DRIs over the years, including calcium and vitamin D, and more recently sodium and potassium ^1, 3^. In contrast, the WHO NRVs have had updates only in sodium and potassium, as well as in calcium supplementation ^4– 6^. Second, the DRIs and NRVs are similar for most micronutrients in terms of AR (but not UL). A few micronutrients have DRIs but not NRVs (in general or in pregnancy), such as choline, vitamin E, phosphorus ^2, 7– 9^.

Nutrient reference values are especially important during pregnancy. There is a biological imperative for increased nutrient intake, and there are well-documented maternal and fetal consequences of inadequate nutrition during the periconceptional, pregnancy, and postpartum periods ^10^. Further, NRVs in pregnancy inform the composition of “prenatal vitamins”, or multiple micronutrient supplements (MMS), used in pregnancy to promote positive pregnancy outcomes ^11^. The commonly used UNIMMAP supplement for pregnancy includes 15 vitamins and minerals at the level recommended by the DRIs.

Yet, there is growing recognition of the limited extent to which the DRI study populations represent all sub-populations, including pregnant and lactating women ^1^. Women have historically been underrepresented in medical research. For example, drug trials to date have been conducted almost exclusively in young men ^12^, and a recent review of clinical trials (including nutraceutical) in India found that less than 2% included pregnant women ^13^. This is problematic because there is likely to be sexual dimorphism in the physiology, metabolism, and related toxicity and efficacy of supplements and drugs. Further, there are clear ethical imperatives to include pregnant women in research because women deserve access to well-studied interventions ^14^. We hypothesize that nutrition science and the related evidence-based guidelines have similarly suffered from excluding women and pregnant women from research.

Meta-research, or meta-science, is an evolving field focused on systematic evaluation of the way science is produced and used ^15^. There are five broad areas of focus for meta-science including: the way research is performing, or methods; the way people communicate about research, or reporting; the way we verify research, or reproducibility; the way we conduct peer review, or evaluation; and the way we reward research, or incentives ^15^. Meta-research is well suited to measure whether specific subpopulations are excluded from biomedical research and whether novel research methods are equally applied to problems or conditions that affect women, underrepresented minority populations, and people living in the global south.

### Objectives

We aim to formally review the methods of research studies used to inform the DRIs and identify opportunities to improve how we fund, perform, evaluate, and incorporate nutrition science for improving public health. The objective of this meta-science study is to summarize the *population* and *nutrition-science methods* used in studies informing the nutrient reference values for women, and for pregnant and lactating women.

### Review questions

This review is focused primarily on assessing the population and methods used in the studies informing the development of DRIs:

1. **Population (Women).** To what extent do the studies informing the population average requirement (AR) and safe upper levels of intake (ULs) include female subjects? Is there variation in the subjects’ race or ethnicity, nutritional or anthropometric status, and health or disease status?
2. **Population (Pregnancy and Lactation).** To what extent do the studies informing the population average requirement (AR) and safe upper levels of intake (ULs) include women across the life course, specifically pregnant and lactating people?
3. **Methods (Study Design)**. Do studies utilize best-in-class methods including: controlled feeding studies and/or well-designed intervention and observational cohorts?
4. **Methods (Molecular)**: Do studies assess functional and metabolic outcomes? Do studies utilize integrated approaches, determining alterations in absorption, kinetics and whole-body nutrient losses using mixed methods approaches (e.g. balance designs, isotopes)?
5. **Methods (Modern)**. Do studies utilize modern methods including: metabolomics, proteomics, data analytics, or adaptive and/or target trial methods? Modern omics technologies include liquid chromatography mass spectrometry for metabolite profiling and protein profiling. Modern data analytic approaches include computational, statistical, and deep learning techniques to analyze omics data with high-dimensionality, correlation between features and zero-inflated data.

Secondary research questions include:

1. **Representativeness** (external generalizability) of the study population: To what extent can we generalize the findings from this study population to other populations?

This review study will *not* assess the quality of the research or risk of bias in each study. Nor will it assess whether the interpretation and conclusions of the studies are accurate or appropriate.

## Methods

**Figure 1.**
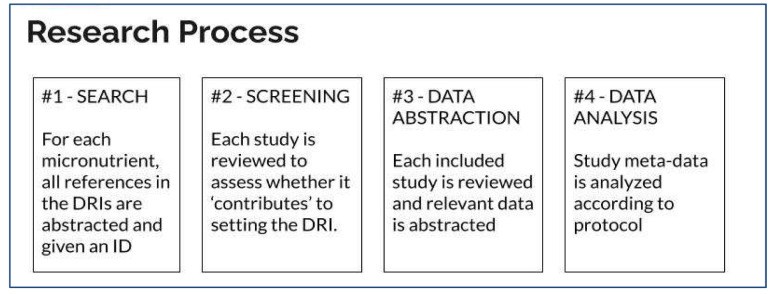
Major components of the meta-review research process.

The research will take part in four phases: 1) search, 2) screening, 3) data abstraction, and 4) data analysis. Figure 1 provides a snapshot of the process.

### Search

The search process will begin with identifying the indicators considered by the committee to establish the DRI. All references in the “Selection of Indicators for Estimating the Requirement for [nutrient]” section will be abstracted. We will also abstract references in the “Findings by Life Stage and Gender Group” Section, specifically for the “Adults”, “Pregnancy”, and “Lactation” sub-sections. Finally, we will abstract references in the “Tolerable Upper Intake Levels” section. The full reference (as listed in the DRI document) and the section in which it appeared will be recorded.

There are two exceptions in the search process requiring slight adjustments to the search strategy. First, for vitamin B2 (riboflavin), an additional section will be included, “Approaches for Deriving the Estimated Average Requirement” because this section provided an overview of B2-related references used to determine the EARs. Second, the latest (2011) calcium and vitamin D report has a different layout of the chapters and sections from other reports. We will abstract references from three chapters in this report: Chapter 4, “Review of Potential Indicators of Adequacy and Selection of Indicators: Calcium and Vitamin D” (Tables 4–8, 4–9, 4–11, 4–12 and the preeclampsia section will be our focus); Chapter 5, “Dietary Reference Intakes for Adequacy: Calcium and Vitamin D”; and Chapter 6, “Tolerable Upper Intake Levels: Calcium and Vitamin D”. To ensure the completeness of the data, we will also abstract references in the “life stages” section of the 1997 calcium and vitamin D report, which determined adequate intakes (AIs) but not estimated average requirements (EARs) for these two nutrients (EARs were set in the 2011 report). 

The following five documents will be reviewed during the search:

1. Institute of Medicine (IOM) 1998. Dietary Reference Intakes for Thiamin, Riboflavin, Niacin, Vitamin B6, Folate, Vitamin B12, Pantothenic Acid, Biotin, and Choline. Washington, DC: The National Academies Press.
2. IOM 2000. Dietary Reference Intakes for Vitamin C, Vitamin E, Selenium, and Carotenoids. Washington, DC: The National Academies Press.
3. IOM 2001. Dietary Reference Intakes for Vitamin A, Vitamin K, Arsenic, Boron, Chromium, Copper, Iodine, Iron, Manganese, Molybdenum, Nickel, Silicon, Vanadium, and Zinc. Washington, DC: The National Academies Press. ( *We will focus on Vitamin A, Vitamin K, Iodine, Iron, and Zinc in this report*)
4. IOM 2011. Dietary Reference Intakes for Calcium and Vitamin D. Washington, DC: The National Academies Press.
5. IOM 1997. Dietary Reference Intakes for Calcium, Phosphorus, Magnesium, Vitamin D, and Fluoride. Washington, DC: The National Academies Press. ( *We will focus on Phosphorus and Magnesium in this report; we will also extract references for calcium and vitamin D to supplement the 2011 report references*)

Three documents will not be reviewed, given that the focus of this meta-research is micronutrient DRIs:

1. National Academies of Sciences, Engineering, and Medicine 2019. Dietary Reference Intakes for Sodium and Potassium. Washington, DC: The National Academies Press.
2. IOM 2005. Dietary Reference Intakes for Water, Potassium, Sodium, Chloride, and Sulfate. Washington, DC: The National Academies Press.
3. IOM 2005. Dietary Reference Intakes for Energy, Carbohydrate, Fiber, Fat, Fatty Acids, Cholesterol, Protein, and Amino Acids. Washington, DC: The National Academies Press.

### Screening

Two researchers will be assigned to each nutrient list and will independently screen the references: one researcher as the main screener and the other for quality control. The rapid screening phase will involve reviewing the DRI document and the study title to determine whether the study meets the inclusion criteria for the study. The product of this screening step will be a list of final records for full-text review and data extraction. 

***Inclusion criteria***

1. The study was used to identify an appropriate indicator to set the DRI.
2. The study was used to inform the determination of current DRI values.
3. The study includes data about at least one of the following vitamin or minerals: ****Thiamin, Riboflavin, Niacin, Vitamin B6, Folate, Vitamin B12, Pantothenic Acid, Biotin, Choline, Vitamin C, Vitamin E, Selenium, Carotenoids, Vitamin A, Vitamin K, Copper, Iodine, Iron, Zinc, Calcium Vitamin D, Phosphorus, and Magnesium ^1^.
4. The study presents primary data (and is not a meta-analysis or modeling study)

***Exclusion criteria***

1. Studies for indicator selection: exclude the reference if a particular indicator was mentioned in the DRI report, but was not used to determine EAR/AIs. ( *e.g., in the Thiamin chapter, a few indicators were noted as “None of these was judged to be a dependable criterion of thiamin status*).
2. Other studies: exclude the reference if this study was mentioned in the report for a particular life stage / UL determination, but with additional comment about it not being used in setting the final DRIs. ( *e.g., in the Thiamin chapter, pregnancy section, a few studies were noted as “Data from the studies cited above are equivocal about the effects of pregnancy on thiamin requirements, and thus are not useful in refining this estimate”*)
3. If the UL cannot be determined for this nutrient, all references in the UL section will be excluded.

### Data abstraction

We will abstract data for all studies that pass through the screening phase. Data will be entered in a study-specific data extraction form, and the following data will be collected.

***Administrative information, study identification***

- Which micronutrient is this study in regard to?
- Which section and subsection of the DRI report chapter was this reference cited?
  - Indicators (subsections include the name of the indicators)
  - Life stages (subsections include adulthood, pregnancy, lactation, and related human milk sections)
  - UL determination (subsections include hazard identification, dose-response assessment, intake assessment, and risk characterization)
- Study ID (Last name of the first author, followed by year, e.g., Smith 2010)
- Full reference (original style as cited in the report)
- Funding source(s)
- Is the article an open-access publication?
- Inclusion/exclusion at full-text stage, reasons for exclusion include:
  - Article cannot be found (through direct online search or through interlibrary loan request)
  - This is a review article and do not provide primary data to help determine DRIs

***Study methods***

- Type of study population: is this a human and/or non-human study? (if *in vitro* study, end of data collection, e.g., cell, organ, tissue)
- What is the design of study?
  - Randomized controlled trial
  - Quasi-experimental study (e.g., treatment study without randomization)
  - Cross-over trial
  - Cohort study
  - Case-control study
  - Cross-sectional study
  - Case report
  - Modeling, kinetics, and other secondary data analysis study
- Describe the study design briefly
  - What was the intervention / exposure / status?
  - Is there a control group? (If yes, brief description of the control)
  - Any outcome pertaining to women’s health? (if yes, brief description of the outcome)
- Rigorous design:
  - Is this a controlled feeding study?
  - Is this a randomized controlled trial?
  - Does this study have repeated measurements on the same participants / subjects over time? (longitudinal design)
- Molecular design:
  - Does this study use stable isotope?
  - Is this a balance study (measured input and excretion)?
  - Does this study measure biomarkers for micronutrient status?
- Modern design:
  - Does the study use any “-omics” method?

***Human population***

- Where was the study conducted? (Country where study population resides)
- Is this a healthy population?
- Does this population share a common condition, comorbidity, or other characteristics? (if yes, describe the shared condition, e.g., all parenteral nutrition, all elderly patients)
- Total sample size
- Are women included in the study population? (If yes, # of females included)
- Are pregnant or lactating people included in the study population? (If yes, # of pregnant or lactating people included)
- What was the average/median age of the population (year)?
  - Were women of reproductive age included (14-45y)?
- Is race/ethnicity reported? (if applicable, indicate # of people identified by each race/ethnicity)

***Non-human subjects***

- Species/breed if animal study
- Total sample sizes (n)
- Are female subjects included? (If yes, Total # females included)
- Are pregnant or lactating animals included? (If yes, Total # pregnant or lactating animals included)
- Is this a special group of subjects? (If yes, describe the shared condition, e.g., gene-knockout animal model, animals with shared comorbidities, all aged or all young animals, etc.)
- Other information relevant to our review

### Analysis plan

After the completion of full-text data extraction, qualitative synthesis will be conducted for all included studies, separately for each micronutrient (and combined for all selected nutrients as needed). The synthesis will prioritize the research questions in terms of population characteristics and methods used in the included studies. We will also import the cleaned dataset into a statistical software (R version 4.0.4, R Core Team, Vienna, Austria) for analysis ^16^. We will conduct univariate descriptive analysis (e.g., summarizing number of included studies for each nutrient); between-group comparisons (e.g., to compare findings between two nutrients, or between two sub-groups), including the Student’s *t*-test (or non-parametric test such as the Mann-Whitney *U* test), chi-squared test; as well as trend test (e.g., to examine annual trends of the publications for individual and all included nutrients).

- **Population (Women).**
  - % women overall and refs in indicator, AR, UL sections
  - % women of reproductive age?
  - % URM overall and refs in indicator, AR, UL sections
  - % low / high BMI overall?
  - % healthy subjects overall
- **Population (Pregnancy and Lactation).**
  - % pregnant women/animals overall and refs in indicator, AR, UL sections
  - % lactating women/animals overall and refs in indicator, AR, UL sections
- **Methods (Rigorous Design)**.
  - % studies used controlled feeding in indicator, AR, UL sections
  - % studies used randomized controlled trials in indicator, AR, UL sections
  - % studies had well-designed interventions in indicator, AR, UL sections
  - % studies were observational cohorts
- **Methods (Molecular Design)**:
  - % studies used stable isotope methods in indicator, AR, UL sections
  - % studies measured biomarkers for micronutrient status in indicator, AR, UL sections
- **Methods (Modern Design)**.
  - % studies used any -omics methods in indicator, AR, UL sections

### Dissemination plan

We will publish the paper in an open access scientific journal. Depending on the amount of evidence we eventually review and generate, there may be more than one manuscript as a result of this project. We will upload our primary (raw) data upon completion of the review to an approved online repository (e.g., Figshare or Harvard Dataverse). We will then share the repository DOI with the manuscript.

### Proposed timeline

**Figure 2.**
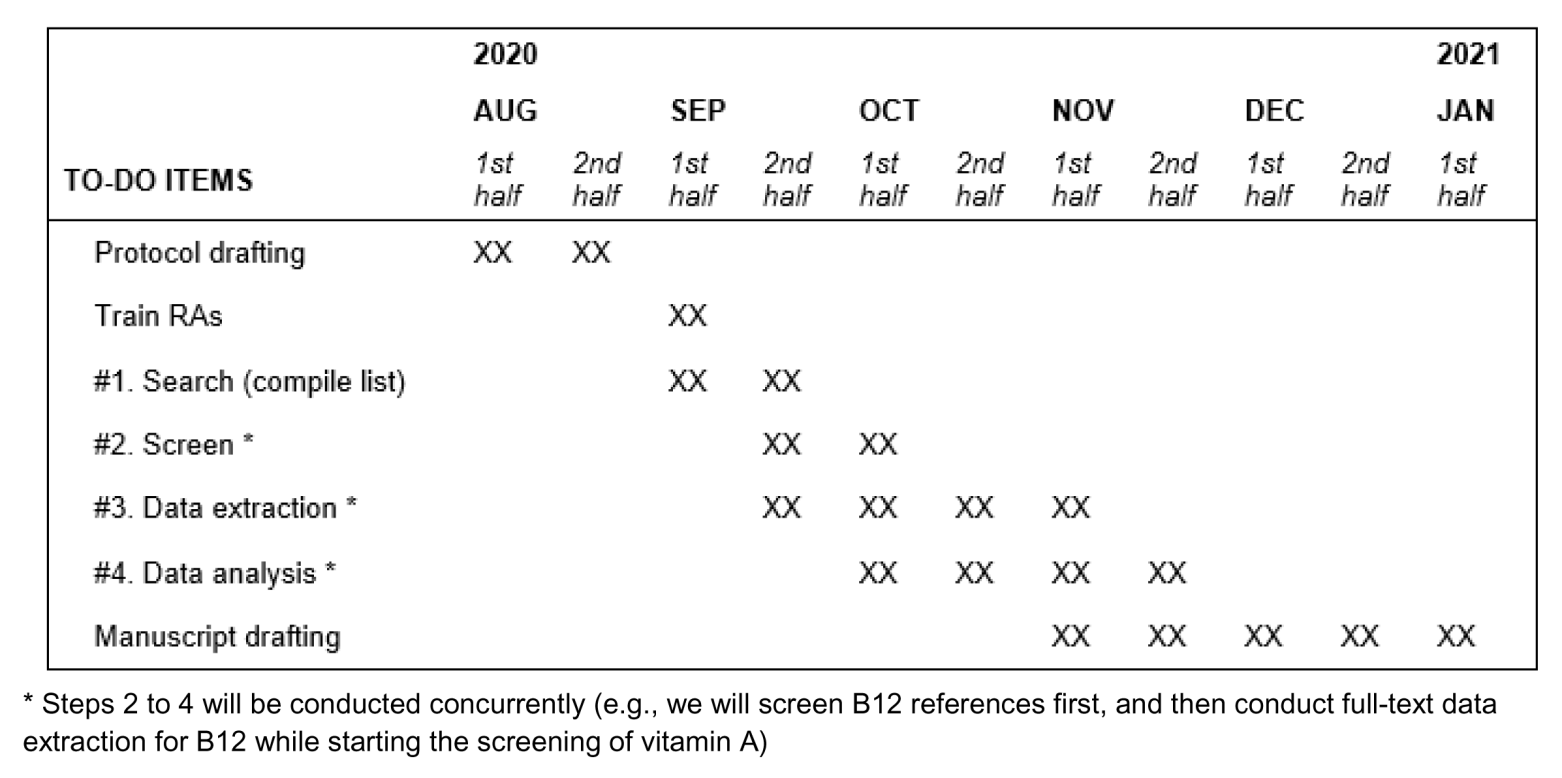
Proposed timeline of the meta-review. * Steps 2 to 4 will be conducted concurrently (e.g., we will screen B12 references first, and then conduct full-text data extraction for B12 while starting the screening of vitamin A)

Figure 2 shows the proposed timeline of the study. At time of publication, we have now completed the compiling of references (pulled citations from the DRI reports), and are currently screening the references. In addition, we have pilot-tested the entire review process for one micronutrient (vitamin B12).

## Data availability

### Underlying data

No underlying data are associated with this article.

## Notes

^1^We excluded several nutrients from the proposed meta-review for the following reasons: i) not a micronutrient, including water, energy, carbohydrate, fiber, fat, fatty acids, cholesterol, protein, and amino acids; ii) an estimated average requirement (EAR) or adequate intake (AI) was not set for a given nutrient, including arsenic, boron, nickel, silicon, vanadium; iii) the UL is not determinable in current DRI, owing to lack of data of adverse effects, and concern regarding lack of ability to handle excess amount, including arsenic, chromium, silicon, sulfate, vanadium; iv) source of intake should be from food only to prevent high levels of intake, including arsenic, chromium, silicon, sulfate, vanadium; and v) not determinable owing to a lack of data of a specific toxicological adverse effect, including sodium and potassium.
