# Supplementary material for: Protocol for meta-research on the evidence informing micronutrient dietary reference intakes for pregnant and lactating women
Source: Gates Open Res. 2020 Nov 13;4:171. [Version 1] doi: 10.12688/gatesopenres.13199.1 (PMC7876347; doi:10.12688/gatesopenres.13199.1)
Supplement: Peer review history

# Peer review: Reviewer response for version 1

**DOI:** 10.21956/gatesopenres.14407.r30259

**Published:** 2021-02-10

**Authors:** Sofa Rahmannia

**Affiliations:** 1 Faculty of Medicine, Universitas Pasundan, Bandung, Indonesia

This study reminds us to re-evaluate existing recommendations along with developments in science and technology. Moreover, DRI is the foundation for determining intervention plans, evaluations, and policies in various sectors. The steps the authors took were excellent, clear, and replicable in other populations and age groups.

 In Phase #2: Screening, the authors may explain what the second researcher will do as a quality controller. It would be better if the two researchers screened simultaneously and matched the results; if there were differences, it could be directly discussed or invited a third researcher. If possible, include the name (with initials) of the investigator conducting the screening.

 In Phase #3: Data Abstraction, the authors may assess the reference used to observe the absorption/bioavailability. Whether it is enough with a balance study (measured input and excretion) only, or is there a reference to observe the micronutrient levels biomarker in circulation.

 This study focuses on women, pregnancy, and lactation. However, for this population, especially in low- and middle-income countries, it is encouraged to note the possibility of pregnancy and lactation in the adolescents' population. Therefore, it is recommended to be considered by the team, primarily if it is found in Phase #3 Data Abstraction.

 The authors are also suggested to elaborate on dietary reference values on specific nutrients from other sources such as Hotz (2007 ^1^) and Arimond *et al. *(2011 ^2^).

Is the study design appropriate for the research question?

Yes

Is the rationale for, and objectives of, the study clearly described?

Yes

Are sufficient details of the methods provided to allow replication by others?

Yes

Are the datasets clearly presented in a useable and accessible format?

Not applicable

Reviewer Expertise:

Maternal and Child Nutrition | Dietary intake

I confirm that I have read this submission and believe that I have an appropriate level of expertise to confirm that it is of an acceptable scientific standard.

## References

[1] : Dietary indicators for assessing the adequacy of population zinc intakes. *Food Nutr Bull*.2007;28(3 Suppl) : 10.1177/15648265070283S304 S430-53 10.1177/15648265070283S304 17988006

[2] : Dietary Diversity as a Measure of Micronutrient Adequacy of Women’s Diets in Resource-Poor Areas. *Fanta 2-Bridge*.2011; Reference source

# Peer review: Reviewer response for version 1

**DOI:** 10.21956/gatesopenres.14407.r30260

**Published:** 2021-02-04

**Authors:** Regan L. Bailey

**Affiliations:** 1 Department of Nutrition Science, Purdue University, West Lafayette, IN, USA

This work summarizes, using a meta-research approach, how nutrient reference intakes were established for pregnant and lactating women in the North American context (i.e. The Dietary Reference Intakes), and is an important contribution to the literature. The authors are to be applauded for pre-publishing efforts a priori to review. The work plan is well organized, clearly written, and scientifically sound.

 Please clarify, is the AR or EAR being used in the global context to harmonize with WHO/FAO, and if the UL is specifically the Tolerable Upper Intake Level or used in the global context?

 Meta-research may be new to some readers, if possible consider expanding the last paragraph of the introduction.

 Consider inviting FNB staff to collaborate (suggest Anne Yaktine as a first point of contact), they are a tremendous resource and will have knowledge not found in the reference section of the reports.

 Assuming inclusion criteria refers to all forms of the micronutrients listed, if not clarify.

 You may wish to examine the evidence scanning paper by Brannon *et al.* (2016 ^1^). Efforts are being made to scan the literature to trigger if a DRI should be updated.

Is the study design appropriate for the research question?

Yes

Is the rationale for, and objectives of, the study clearly described?

Yes

Are sufficient details of the methods provided to allow replication by others?

Yes

Are the datasets clearly presented in a useable and accessible format?

Not applicable

Reviewer Expertise:

Dietary assessment, nutrition epidemiology, NHANES, dietary supplements, precision nutrition

I confirm that I have read this submission and believe that I have an appropriate level of expertise to confirm that it is of an acceptable scientific standard.

## References

[1] : Scanning for new evidence to prioritize updates to the Dietary Reference Intakes: case studies for thiamin and phosphorus. *Am J Clin Nutr*.104(5) : 10.3945/ajcn.115.128256 1366-1377 10.3945/ajcn.115.128256 27733406
